# The non-conveyance of trauma patients in Swedish emergency medical services: a retrospective observational study of the trauma population not transported to an emergency department

**DOI:** 10.1186/s12873-024-00952-9

**Published:** 2024-02-27

**Authors:** Glenn Larsson, Jana Eldh, Elisabeth Hagman, Magnus Andersson Hagiwara

**Affiliations:** 1 Centre for Prehospital Research, Faculty of Caring Science, Work Life and Social Welfare, University of Borås, SE-501 90 Borås, Sweden; 2 Department of Prehospital Emergency Care, Sahlgrenska University Hospital, Gothenburg, Sweden; 3 PICTA, Prehospital Innovation arena, Lindholmen Science Park, Gothenburg, Sweden; 4 Faculty of Health and Society, Department of Care Science, Malmö University, SE-205 06 Malmö, Sweden

**Keywords:** Emergency medical services, Triage, Non-conveyance, Trauma patient, Ambulance

## Abstract

**Introduction:**

Due to a systemic modification in Swedish emergency medical services (EMS) staffing in recent years, the nature of the Swedish EMS has changed. Transport to an emergency department (ED) is no longer the only option. Referrals and non-conveyance form a growing part of EMS assignments. Trauma is one of the most common causes of death and accounts for 17% of Swedish EMS assignments. The aim of this study was to describe the characteristics and clinical outcomes of non-conveyed trauma patients who were assessed, treated and triaged by the EMS to gain a better understanding of, and to optimise, transport and treatment decisions.

**Methods:**

The study had a descriptive, retrospective and epidemiologic design and was conducted by reviewing EMS and hospital records for 837 non-conveyed trauma patients in the southwest of Sweden in 2019.

**Results:**

Three in four non-conveyed trauma patients did not seek further medical care within 72 h following EMS assessment. The patients who were admitted to hospital later were often older, had suffered a fall and had a medical history. Half of all the incidents occurred in a domestic environment, and head trauma was the major complaint. Less than 1% of the studied patients died.

**Conclusion:**

Most of the non-conveyed trauma patients did not seek further medical care after being discharged at the scene. Falling was the most common trauma event, and for the older population, this meant a higher risk of hospital admission. The reasons for falls should therefore be investigated thoroughly prior to non-conveyance decisions. Future studies should focus on the reasons for non-conveyance and measure the morbidity and invalidity outcomes rather than mortality.

## Introduction

Due to a systemic modification in Swedish emergency medical services (EMS) staffing in recent years, the nature of the Swedish EMS has changed. With the introduction of specialist registered nurses (RNs) in the organisation, the care has gradually become more advanced; the EMS has moved from being a load-and-go transport organisation to becoming an integral part of the healthcare system. Where the emergency department (ED) was previously the default destination for EMS patients, a mandate now exists to triage patients to other care options (e.g. self-care, primary care centres [PCCs]), where possible, or directly to definitive care via a suitable Prehospital Fast Track Care (PFTC) program [1]. This means that non-conveyed patients (i.e. patients not transported to an ED by the EMS) are becoming more common [2]. A study in the Netherlands showed that one in three ambulance runs ended in non-conveyance, and of these, half were referred to a PCC or ED by means other than an ambulance [3]. Similarly, a study found that 14% of EMS patients in Sweden were not conveyed to a healthcare facility [4].

Trauma patients are common in the EMS and form a heterogenous group with minor to severe injuries. Trauma is one of the most common causes of death, especially among young people, and accounts for 17% of Swedish EMS assignments. While traffic-related deaths are declining, an increase in low-energy fall trauma, mainly among the elderly, has been seen in recent years [5].

The majority of trauma patients in Sweden are transported to an ED, and severely injured patients are well described in the national trauma registry [6]. However, a considerable proportion of patients are triaged to a PCC or remain at the scene and receive advice on self-care [1]. Trauma accounts for between 22% and 24% of all EMS non-conveyance assignments [3, 7]. However, little is known about non-conveyed trauma patients and the outcomes of EMS decisions. Gaining knowledge is the first step in enhancing the quality of care as the EMS continues to evolve to meet future demands. The aim of this study was therefore to describe the EMS assessments, treatments and outcomes of the non-conveyed trauma population.

## Methods

### Design

The study had a descriptive, retrospective and epidemiologic design and was conducted by reviewing EMS and hospital records. A STROBE checklist was used to enhance the quality of the report [8].

### Study setting and population

The study was conducted in 2019 in the southwest of Sweden in a region with 1.7 million inhabitants and 173,536 ambulance assignments in the same year. The EMS in the region consists of around 110 ambulance units at 46 stations divided into five hospital organisations [9]. The region has 10 hospitals with EDs [10].

Ambulances in Sweden are staffed with two individuals: at least one RN, usually with a postgraduate education in prehospital emergency care or a similar field, and an emergency medical technician (EMT). An EMT in Sweden is an assistant nurse with 40-week specialist training in prehospital emergency care [1]. EMS personnel work according to regional guidelines, which describe the patient assessments, treatments and care for various conditions. RNs also have pharmaceuticals that they are able to administer [11].

The Rapid Emergency Triage and Treatment System (RETTS) is a triage tool used in the majority of the Swedish EMS [12]. It comprises two parts: vital signs and presented symptoms or conditions. Together, a coloured emergency signs and symptoms (ESS) code is assigned to describe the condition and a priority colour to indicate its urgency. Red is construed as life-threatening, followed by orange (potentially life-threatening), yellow and green (can wait without medical risk) [12].

### Data sampling

**Fig. 1:**
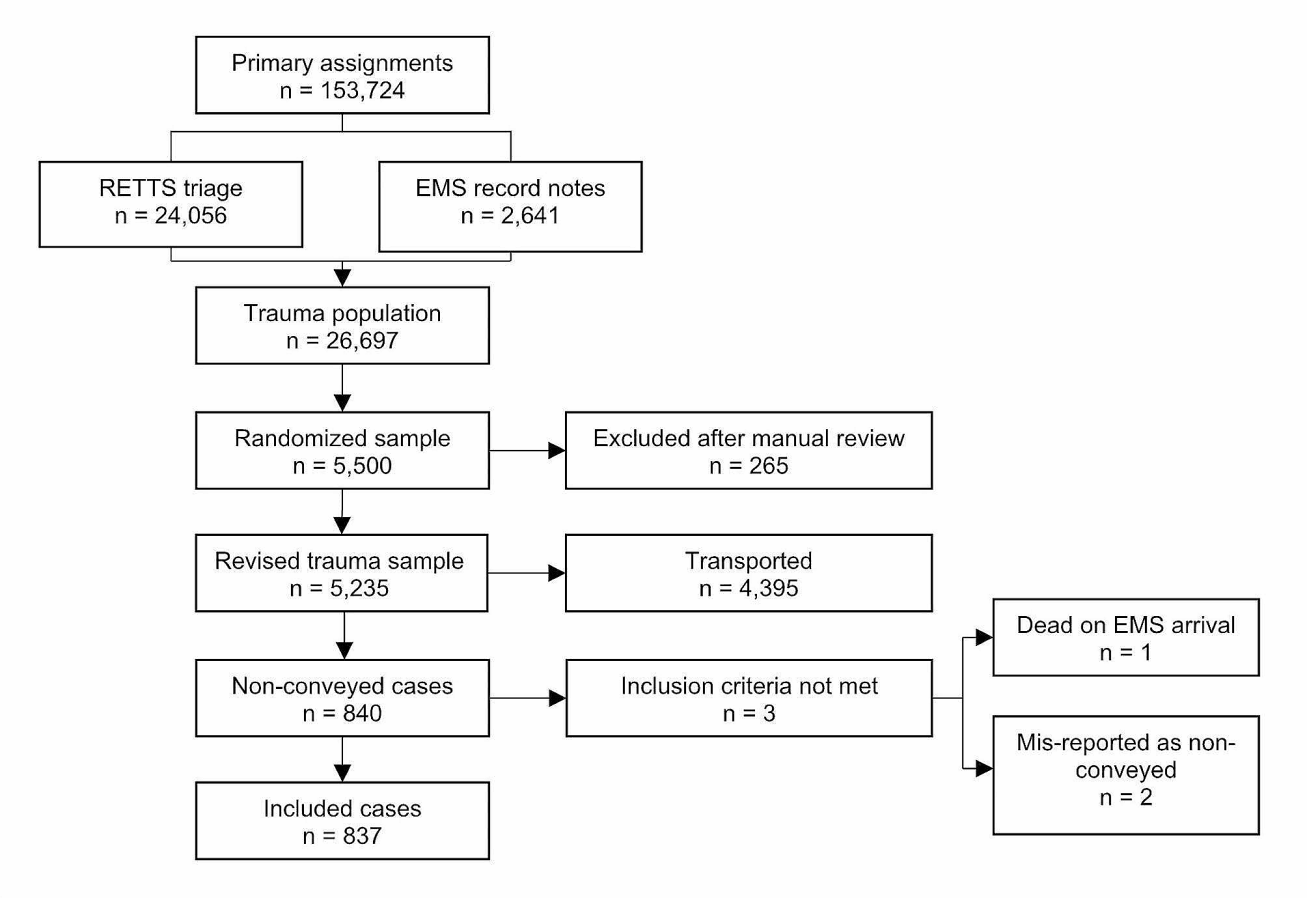
Flow chart of sample selection

The data were collected retrospectively from 1 January to 31 December 2019. A total of 153,724 primary EMS assignments were conducted during this period. Of these, 24,056 were registered with an ESS code to indicate physical trauma or injury (Fig. 1), and 2,641 records were registered without an ESS code. The latter records were initially included and read to not miss any potentially time-critical patients. This provided a total of 26,697 assignments (17.4%). Of these, 5,500 cases were randomised based on assignments, with proportional distribution for each of the five organisations, which provided a representative sample of the trauma population. After a manual review by designated ambulance RNs, 265 cases were excluded for the following reasons: inter-hospital transport, missing EMS record, missing personal identification number, secondary transport assignment or not trauma-related. Of the remaining 5,235 records, 840 patients were non-conveyed. A further review resulted in the exclusion of three assignments. In these cases, the patients were found to be either deceased upon EMS arrival or had been misreported as non-conveyed (Fig. 1). This gave a final sample of 837 patients who met the inclusion criteria of having suffered physical trauma or injury, been seen by the EMS and not been transported to an ED by the EMS.

### Patient record review

The electronic EMS patient records provided data on the time, sex, age, EMS dispatch priority, mechanism of injury, location of injury, vital signs, RETTS priority, ESS code, EMS treatment, type of transport, destination (remain at scene/PCC) and additional ambulance assignments within 72 h. The hospitals’ electronic patient records provided information on the patients’ past medical histories (previous diagnoses assessed by a physician), in-hospital examinations, treatments, diagnose codes (International Classification of Diseases 10th revision [ICD-10]), length of hospital stay and intensive care (IC) treatment. The diagnostic code, treatment and examination data were used to determine which patients had visited the ED and/or been admitted to hospital within 72 h. The definition for visits to the ED was that the patient went home after the ED visit. Admitted patients were admitted to one of the hospital’s departments. The ICD-10 codes were grouped into categories (type of complaint) to enhance the presentation. The mortality variables were retrieved from the Swedish population register.

### Data analysis

The outcome data were summarised using descriptive statistics. The numbers and proportions are presented as numbers and percentages, medians and interquartile range. Group comparisons (all non-conveyed, primary care referral, ED visit, hospital admission) were performed using Pearson’s chi-square test or the Fisher–Freeman–Halton exact test, where applicable. The medians were compared using the Mann–Whitney *U* test and Kruskal–Wallis *H* test. All the tests were two-sided, and *p*-values below 0.01 were considered significant. IBM SPSS Statistics version 28.0.1.1 was used for the statistical analyses.

### Ethics

This study was approved by the Swedish Ethical Review Authority in Stockholm, Sweden (Dnr 2020 − 00490). Approval was given by the heads of operations of the concerned organisations. The need for written informed consent was waived by the Swedish Ethical Review Authority in Stockholm, Sweden due to retrospective nature of the study. The designated reviewers were employees of the respective organisations. Strict compliance with Swedish research ethics guidelines was upheld throughout.

## Results

Of the 5,235 trauma cases, 837 (16%) of the patients (age range, 0–102 years) were not transported to an ED. Among them, 47% were women, and children (0–17 years) accounted for 17% of the total population. The largest age group (25%) was 18–39 years, followed by patients aged 40–64 years (22%) and 65–79 years (19%). The median age was 48 years, with a statistically significant difference between men and women. The median age for the patients admitted to hospital was significantly higher than that of the total patient population. Patients who reported no pre-existing medical conditions made up 492 (59%) of the total. The most common conditions among the patients were *circulatory conditions* (22%), followed by *psychiatric conditions* (16%). *Circulatory conditions* and *other illnesses* were associated with hospital admissions. Two fifths of the EMS assignments were carried out during normal work hours (08.00–17.00), and 95% of the EMS dispatches were priority 1 and 2 (Table 1).

**Table 1:** Patient characteristics and outcome on visiting ED and hospital admission within 72 h, n (%)

		All non- conveyed	*p*-value	Primary Care Referral	*p*-value^1^	ED Visit	*p*-value^1^	Hospital Admission	*p*-value^1^
**Total**		837 (100)		132 (100)		136 (100)		44 (100)	
**Sex**	Male	419 (50)		70 (53)	*0,096*	76 (56)	*0,045*	23 (52)	*0,823*
	Female	397 (47)		62 (47)		60 (44)		21 (48)	
	Missing	21 (3)		0 (0)		0 (0)		0 (0)	
**Age Median (Q1, Q3)**		48 (22, 74)		56 (27, 79)	*0,026*	52 (28, 78)	*0,051*	76 (51, 84)	< 0,001*
**Age/Sex**	Male	41 (21, 70)	< 0,001*	51 (21, 76)	*0,069*	51 (27, 78)	*0,381*	70 (45, 81)	*0,204*
	Female	52 (28, 78)		58 (32, 82)		55 (28, 79)		79 (66, 86)	
**Age Group**	0–17	142 (17)		17 (13)	*0,078*	13 (10)	*0,031*	3 (7)	< 0,001*
	18–39	210 (25)		33 (25)		41 (30)		5 (11)	
	40–64	181 (22)		22 (17)		25 (18)		6 (14)	
	65–79	155 (19)		32 (24)		31 (23)		15 (34)	
	80+	135 (16)		28 (21)		26 (19)		15 (34)	
	Missing	14 (2)		0 (0)		0 (0)		0 (0)	
**Time of Dispatch**	08:00–17:00	358 (43)		94 (71)	< 0,001*	66 (49)	*0,090*	25 (57)	*0,184*
	17:00–00:00	320 (38)		27 (20)		53 (39)		13 (30)	
	00:00–08:00	157 (19)		10 (8)		17 (13)		6 (14)	
**EMS Dispatch Priority**	1	273 (33)		43 (33)	*0,188*	32 (24)	*0,038*	7 (16)	*0,022*
	2	521 (62)		78 (59)		97 (71)		33 (75)	
	3	43 (5)		11 (8)		7 (5)		4 (9)	
**Medical History**	No prior illness	492 (59)		82 (62)	*0,685*	66 (49)	*0,003*	14 (32)	< 0,001*
	Circulatory	187 (22)		38 (29)	*0,110*	40 (29)	*0,104*	21 (48)	< 0,001*
	Diabetes	52 (6)		15 (11)	*0,024*	11 (8)	*0,536*	4 (9)	*0,492*
	Pulmonary	48 (6)		5 (4)	*0,298*	15 (11)	*0,022*	7 (16)	*0,021*
	Psychiatric	131 (16)		15 (11)	*0,136*	25 (18)	*0,572*	11 (25)	*0,210*
	Other	279 (33)		43 (33)	*0,469*	61 (45)	*0,010*	28 (64)	< 0,001*
	Missing	66 (8)		7 (5)		9 (7)		2 (5)	
**Mechanism of Injury**	Traffic	157 (19)		22 (17)	*0,812*	20 (15)	*0,585*	0 (0)	< 0,001*
	Violence	123 (15)		17 (13)		18 (13)		4 (9)	
	Fall	386 (46)		65 (49)		67 (49)		28 (64)	
	Other	156 (19)		24 (18)		26 (19)		10 (23)	
	Missing	15 (2)		4 (3)		5 (4)		2 (5)	
**Location**	Domestic	428 (51)		62 (47)	*0,342*	64 (47)	*0,539*	28 (64)	*0,025*
	School/Work	24 (3)		5 (4)		5 (4)		0 (0)	
	Public	293 (35)		46 (35)		46 (34)		6 (14)	
	Leisure	42 (5)		8 (6)		10 (7)		3 (7)	
	Other	16 (2)		0 (0)		2 (1)		1 (2)	
	Missing	37 (4)		11 (8)		10 (7)		6 (14)	

^1^*p*-value for categories calculated using Fisher-Freeman-Halton Exact Test. *P*-value for medians calculated using Mann-Whitney U Test. *p* < 0,001 was considered statistically significant.

^2^*p*-value calculated using Kruskal-Wallis H Test.

### Mechanism of injury

Falling accounted for 46% of the cases and was associated with hospital admission. Of the 386 patients who sustained a fall, 69% had fallen in a domestic environment, which made falling at home the most common incident in the non-conveyed trauma population (*p* < 0.001). The fall trauma patients made up 49% of the PCC referrals and ED visits, respectively, and 64% of the hospital admissions (Table 1) out of these 24 had previous circulatory disease (35.8%), 5 had diabetes (7.5%), 9 had lung disease (13.4%), 15 had psychiatric illness (22.4%) and 36 had other diseases (53.7%) Among the younger adults (18–39 years), traffic- and violence-related trauma were the most frequent injuries at 34% and 28%, respectively.^1^ Younger adults were also more likely to suffer trauma in a public place (53%) (not included in Table 1).

### EMS assessment and treatment

Patient assessments (A-E) were conducted in 662 (79%) of the cases, and pain was reported in 40% of the patients (Table 2). On-scene EMS treatments for this population included fracture splinting (prior to referring the patient to the ED by transport other than EMS), pressure dressings and pharmaceutic treatment (mainly analgesia). Eight patients were given sedatives (benzodiazepine). *Other drugs* were given to 8% of the patients. This category included a variety of medications not primarily associated with trauma care. Pressure dressings were applied in 32 (4%) patients. One patient had an on-scene epileptic seizure and needed temporary airway management (not included in Table 2).

**Table 2:** EMS assessment, treatment, and outcome, n (%)

			All non-conveyed	Primary Care Referral	*p*-value^1^	ED Visit	*p*-value^1^	Hospital Admission	*p*-value^1^
Total			837 (100)	132 (100)		136 (100)		44 (100)	
**A-E** **Assess-ment**	**No**		156 (19)	26 (20)	*0,140*	26 (19)	*0,733*	9 (20)	*0,772*
	**Yes**		662 (79)	106 (80)		106 (78)		35 (80)	
	**Missing**		19 (2)	0 (0)		4 (3)		0 (0)	
**Pain**	**No**		436 (52)	52 (39)	*0,005*	50 (37)	< 0,001*	13 (30)	*0,002*
	**Yes**		337 (40)	69 (52)		75 (55)		29 (66)	
	**Missing**		64 (8)	11 (8)		11 (8)		2 (5)	
**ESS Code**	**Adults**	30 Head	218 (26)	1 (0,8)	< 0,001* ^*2*^	34 (25)	*0,007*	9 (20)	< 0,001* ^*2*^
		31 Torso/Genitals	68 [8]	1 (0,8)		19 (14)		9 (20)	
		33 Upper Extremity	39 (5)	2 (2)		11 (8)		4 (9)	
		34 Lower Extremity	96 (11)	1 (0,8)		26 (19)		11 (25)	
		35 Burns etc.	18 (2)	0 (0)		2 (1)		0 (0)	
		36 Drowning	1 (0,1)	0 (0)		0 (0)		0 (0)	
		37 Eye	10 (1)	0 (0)		0 (0)		0 (0)	
		38 Trauma Algorithm	39 (5)	0 (0)		3 (2)		0 (0)	
		41 Animal bites/Stings	17 (2)	1 (0,8)		1 (0,7)		0 (0)	
		42 Assault/Sexual	10 (1)	34 (26)		2 (1)		2 (5)	
		53 Non-specific Symptoms	2 (0,2)	19 (14)		0 (0)		0 (0)	
		86 Self-harm	5 (0,6)	11 (8)		2 (0,7)		2 (5)	
	**Children**	130 Head	41 (5)	27 (20)	*0,308*	1 (0,7)	*0,134*	0 (0)	*0,124*
		131 Torso/Genitals	6 (0,7)	2 (2)		1 (0,7)		1 (2)	
		133 Upper Extremity	5 (0,6)	0 (0)		2 (1)		0 (0)	
		134 Lower Extremity	5 (0,6)	0 (0)		1 (0,7)		0 (0)	
		135 Burns etc.	2 (0,2)	3 (2)		0 (0)		0 (0)	
		137 Eye	2 (0,2)	1 (0,8)		0 (0)		0 (0)	
		138 Trauma Algorithm	3 (0,4)	2 (2)		0 (0)		0 (0)	
		141 Animal bites/Stings	1 (0,1)	0 (0)		0 (0)		0 (0)	
		142 Assault/Sexual	1 (0,1)	1 (0,8)		1 (0,7)		0 (0)	
	**Missing**		248 (30)	18 (14)		30 (22)		6 (14)	
**RETTS priority**	**Red**		1 (0,1)	0 (0)	< 0,001*	0 (0)	*0,058*	0 (0)	*0,018*
	**Orange**		54 (6)	14 (11)		12 (9)		7 (16)	
	**Yellow**		118 (14)	29 (22)		26 (19)		7 (16)	
	**Green**		409 (49)	69 (52)		68 (50)		24 (55)	
	**Missing**		255 (30)	20 (15)		30 (22)		6 (14)	
**Treat-ment**	**Fracture stabilization**		5 (0,6)	0 (0)	*1,000*	1 (0,7)	*0,586*	0 (0)	*1,000*
	**Pressure dressing**		32 (4)	2 (2)	*0,212*	4 (3)	*0,806*	0 (0)	*0,171*
	**Medical treatment**		114 (14)	18 (14)	*1,000*	22 (16)	*0,338*	6 (14)	*1,000*
		Opiate	14 (2)	6 (5)	*0,014*	6 (4)	*0,016*	4 (9)	*0,005*
		Non-opiate analgesics	34 (4)	8 (6)	*0,649*	14 (10)	*0,005*	4 (9)	*0,133*
		Sedation	8 (1)	0 (0)	*0,619*	1 (0,7)	*1,000*	0 (0)	*1,000*
		Oxygen	3 (0,4)	2 (2)	*0,067*	0 (0)	*1,000*	0 (0)	*1,000*
		Infusion	3 (0,4)	3 (2)	*0,004*	1 (0,7)	*0,411*	1 (2)	*0,150*
		Other	66 (8)	10 (8)	*1,000*	8 (6)	*0,483*	2 (5)	*0,569*

^1^*p*-value calculated using Fisher-Freeman-Halton Exact Test. Pearson Chi Square Test used for ESS Code calculation. *p* < 0,001 was considered statistically significant

^2^*p* < 0,001 holds true for adult population and total population

### EMS on-scene triage

EMS patient triage was undertaken according to the RETTS, and 70% of the patients were triaged. Among those not triaged, 7% were transported or referred to a PCC, 12% visited an ED, and 2% were admitted to hospital. A total of 132 (16%) patients were referred to a PCC, and 68% of these were transported there by the EMS. The most frequent ESS code was head trauma in both adults and children. Priority was low (green or yellow) in 90.5% of the triaged cases. Of the patients, 605 (72%) had no further contact with a PCC or ED after the primary EMS dispatch, although seven patients received a secondary EMS assignment within the studied time frame.

### Hospital visits

With respect to hospital visits, 136 patients visited an ED within 72 h of the primary EMS assignment. Receiving secondary EMS transport was a statistically significant factor in both ED visits and hospital admissions. One in four patients had been referred to a PCC before visiting the ED. Head injuries accounted for 27% of the cases diagnosed in the ED, followed by fractured and injured extremities at 26%. Meanwhile, 22% of the cases were categorised as *Other*, received a general health survey, or were held for observation that did not result in a diagnosis (Table 3). Among the patients who visited the Emergency Department (ED), there were 65 patients who did not undergo any intervention. Among the others, 10 patients received treatment for fracture or dislocation, 7 received wound care or suturing, 2 received antibiotics, 20 underwent X-rays, 3 underwent surgery, and 18 patients received another type of management.

**Table 3:** Diagnose, median age, comorbidity and outcome for patients seeking hospital care within 72 h of primary EMS assignment; *n* = 136 (%)

		ED visitors	*p*-value	Hospital Admissions	*p*-value	7 days mortality	*p*-value
**Total**		136 (100)		44 (32)		2 (1, 5)	
**72 h Secondary EMS transport**		74 (54)	< 0,001*	28 (21)	< 0,001*	2 (1, 5)	*0,084*
**Other transport**		62 (46)		16 (12)		0 (0)	
**ED Diagnose**	Fracture Extremity	15 (11)	*1,000*	5 (4)	*0,023*	0 (0)	*0,062*
	Fracture Other	8 (6)		3 (2)		0 (0)	
	Injury Extremity	21 (15)		3 (2)		0 (0)	
	Injury Head/spinal	37 (27)		6 (4)		0 (0)	
	Injury Torso	8 (6)		4 (3)		0 (0)	
	Medical	16 (12)		8 (6)		2 (1, 5)	
	Other	29 (21)		13 (10)		0 (0)	
	Missing	2 (1, 5)		2 (1, 5)		0 (0)	
**Median Age (Q1, Q3)**	Fracture Extremity	68 (25, 89)		89 (68, 95)			
	Fracture Other	74 (52. 81)		78 (65, 94)			
	Injury Extremity	31 (22, 65)		86 (31, 88)			
	Injury Head/spinal	55 (21, 78)		81 (71, 85)			
	Injury Torso	44 (23, 71)		49 (16, 75)			
	Medical	65 (43, 77)		62 (48, 77)			
	Other	43 (30, 78)		78 (52, 81)			
	Missing	3**					
		**No prior illness**		**One prior illness**		**Two or more**	**Missing**
**Comorbidity** (***n*** = **135 ED visitors)**	Fracture Extremity	6 (4)		2 (1)		5 (4)	2 (1)
	Fracture Other	2 (1)		2 (1)		4 (3)	0 (0)
	Injury Extremity	11 (8)		2 (1)		6 (4)	2 (1)
	Injury Head/spinal	15 (11)		9 (7)		13 (10)	1 (0,7)
	Injury Torso	3 (2)		3 (2)		2 (1)	0 (0)
	Medical	3 (2)		5 (4)		7 (5)	1 (0,7)
	Other	6 (4)		10 (7)		10 (7)	3 (2)

*p-value calculated using Fisher-Freeman-Halton Exact Test. *p* < 0,001 was considered statistically significant.

** age unknown

Of the patients who visited the ED, one in three patients were admitted to hospital ward, and two received treatment in an IC. The hospital stays among the admitted patients ranged between 1 and 11 days, with a median stay of three days (not included in Table 3).

### Mortality

The mortality rate after 30 days was 0.8% (seven patients), and these patients had a median age of 69 (65.91) years. All these patients had suffered a fall trauma (not included in Table 3). Five patients were assessed by the on-scene EMS as having head trauma (ESS code 30), one died from unknown causes, and one, who was later admitted to hospital, died from heart failure. All the other deaths occurred outside the hospital. One patient, an elderly woman in palliative home care, died within two days of the EMS assignment. Four patients passed away within seven days; one of them had visited the ED but not been admitted. All deceased patients had comorbidities in the form of circulatory diseases, dementia, epilepsy, mental illness, and alcohol abuse.

## Discussion

This study shows that close to three in four non-conveyed trauma patients did not seek further medical care within 72 h following assessment by the EMS. Those who were later admitted to hospital were older, had suffered a fall and were more likely to have a medical history. Half of all the incidents occurred in a domestic environment. Head trauma was the primary complaint in both the adult population and the population as a whole. Less than 1% of the studied patients died. The non-conveyed trauma patients constituted 16% of the full trauma sample. This finding is in line with those of other studies [4, 13], although the lack of studies that exclusively address non-conveyed trauma patients mars the general comparison.

More than half the patients had no prior illnesses, and the most common form of medical history was *circulatory conditions*. When compared with the findings of Magnusson et al. (2020) [14], the relatively high percentage of patients with no prior illnesses in this study can be explained by the fact that this study included children and, as a trauma study, did not include exclusively medical cases.

Breeman et al. (2018) [7] found that one third of the patients in their study sought medical care after not being conveyed by the EMS. This is incongruent with the findings (28%) of this study. It must be noted, though, that this study only presents data for patients who were referred to a PCC, not those who may have visited a PCC independently. A Swedish study showed that 4% of adults and 6% of children had trauma complaints, and of these, 3% of the adults and 5% of the children visited the ED within seven days [13]. This is lower than the results of this and other studies [7, 14]. Based on the results of this study, it is difficult to draw conclusions about the consequences for non-conveyed patients who later sought emergency department (ED) care. This is partly because we do not know if the return visit was correlated with the reasons the patients initially sought emergency medical services (EMS). The same applies to mortality, as the study cannot provide any data on the cause of later hospital deaths. The study indicates that of the patients who were later admitted to the hospital, over 65% were over 65 years old. It is possible that assessment tools and triage systems may not adequately consider age and comorbidity. The triage system RETTS, which was used during the study period, has, in a study conducted in the ED [15], demonstrated increased accuracy in assessing critically ill patients when the system is combined with the age-combined Charlson comorbidity index [16]. Within pre-hospital emergency care, it could also be valuable to combine existing triage systems with instruments that assess frailty.

Bäckström et al. (2018) [5] showed that the prevalence of fall trauma in the elderly has increased in recent years due to the ageing population. Tinetti (2003) [17] ranked falling as the number one mechanism of injury and stated that a third of the population over 65 years fall at least once a year. The present study confirms domestic fall trauma as the most commonplace mechanism of injury, especially among the elderly. It is also worth mentioning that all the deaths in this study, though not very many, were related to falls. Falling is a general mechanism of injury with diverse causes, which can range from leisure activities and workplace accidents to medical reasons, such as dyspnoea, dizziness and poor eyesight. This suggests that the reason a patient falls could sometimes be more important than the trauma itself. EMS personnel therefore need to be careful to also examine the cause of the fall in order not to miss treatable medical conditions, especially when dealing with the older population.

The fact that one third of the patients in the present study were not triaged by the EMS may seem surprising, but in a trauma setting, a full examination and documentation may not always be completed due to patient compliance or the nature of the incident. The majority of the triaged patients were considered lower priority (green or yellow), which seems reasonable due to the fact that higher priority trauma patients would not be eligible for non-conveyance but would be transported to the ED.

### Limitations

The cases in this study were extracted from a randomised selection, which minimises selection bias and is considered a strength of this study. The patient records were manually reviewed by designated RNs who were familiar with the RETTS and the EMS medical record system [18]. One limitation of the study is that the data on ED triage were not available, which could have been beneficial to better evaluate the EMS triage. A further limitation is that the non-conveyed patients were not compared with conveyed patients in a larger sample.

## Conclusions

The results of this study revealed that most non-conveyed trauma patients did not seek further medical care after having been discharged on the scene, which suggests that the EMS triage in many cases was accurate. Falling was the most common trauma event, and for the older population, this meant a higher risk of hospital admission. The reason for falling should therefore be investigated thoroughly prior to the decision on non-conveyance. In future studies, deeper knowledge of patients’ medical histories (e.g. arrythmias, anticoagulant medication) would be of clinical interest. The risk of mortality was low in this patient segment; however, neither morbidity nor invalidity were evaluated in this study, and these may have been more useful outcomes measures. Due to the lack of collected data, it was not possible to describe the reasons for the non-conveyance decisions by the EMS in this study (i.e. whether they were due to the absence of a discernible need for further care, a referral agreement with another form of transport or the patient refusing further treatment). Nevertheless, studies such as this one could be used to improve future guidelines on non-conveyance by EMS.

## Data Availability

No datasets were generated or analysed during the current study.

## Footnotes

1 *p* < 0.001 by Pearson’s chi-square test.

## Abbreviations

ED: Emergency Department
EMS: Emergency Medical Services
PCC: Primary Care Center
IC: Intensive Care
EMT: Emergency Medical Technician
RN: Registered Nurse
RETTS: Rapid Emergency Triage and Treatment System
ESS: Emergency Signs and Symptoms
